# Supplementary material for: Structural mechanism of ligand activation in human calcium-sensing receptor
Source: eLife. 2016 Jul 19;5:e13662. doi: 10.7554/eLife.13662 (PMC4977154; doi:10.7554/eLife.13662)
Supplement: Table 1—source data 1. — DOI: http://dx.doi.org/10.7554/eLife.13662.004 [file elife-13662-table1-data1.doc]

**Table 1 – source data 1. Anomalous data collection**

| Functional State | Inactive (0mM Ca^2+^) | |
| --- | --- | --- |
| Crystal | Form I (0mM Ca^2+^) - 1 | Form I (0mM Ca^2+^) - 2 |
| **Data collection** |  |  |
| Space group | F222 | F222 |
| Wavelength (Å) | 1.7712 | 1.7712 |
| Cell dimensions   |  |  |
| *a*, *b*, *c* (Å) | 126.5, 150.6, 214.6 | 126.6, 150.6, 214.9 |
|     α, β, γ (°) | 90.0, 90.0, 90.0 | 90.0, 90.0, 90.0 |
| Resolution (Å) | 107.3 - 3.8 (4.4 - 3.8) | 107.4 - 3.6 (4.2 - 3.6) |
| *R*_sym_ or *R*_merge_ | 0.140 (0.639) | 0.141 (0.711) |
| *I* / σ*I* | 13.8 (3.9) | 15.3 (3.9) |
| Completeness (%) | 99.8 (99.8) | 99.9 (99.9) |
| Redundancy | 6.9 (6.8) | 6.9 (6.9) |
| No. reflections | 10126 | 11765 |
| Anomalous CC (%) | 14.2 | 9.5 |
| CC_1/2_ (%) | 99.8 (96.4) | 99.8 (94.9) |

| Functional State | Inactive (0mM Ca^2+^) | |
| --- | --- | --- |
| Crystal | Form I (0mM Ca^2+^) - 3 | Form I (0mM Ca^2+^) - 4 |
| **Data collection** |  |  |
| Space group | F222 | F222 |
| Wavelength (Å) | 1.7712 | 1.7712 |
| Cell dimensions   |  |  |
| *a*, *b*, *c* (Å) | 126.5, 150.4, 214.8 | 126.7, 150.5, 214.4 |
|     α, β, γ (°) | 90.0, 90.0, 90.0 | 90.0, 90.0, 90.0 |
| Resolution (Å) | 88.3 - 3.6 (4.2 - 3.6) | 107.2 - 3.8 (4.4 - 3.8) |
| *R*_sym_ or *R*_merge_ | 0.138 (0.847) | 0.135 (0.671) |
| *I* / σ*I* | 15.2 (3.5) | 14.4 (3.7) |
| Completeness (%) | 99.9 (100.0) | 99.9 (99.9) |
| Redundancy | 6.9 (7.0) | 6.9 (6.8) |
| No. reflections | 11672 | 10120 |
| Anomalous CC (%) | 10.7 | 11.0 |
| CC_1/2_ (%) | 99.9 (95.2) | 99.9 (94.7) |

| Ligand-binding State | Inactive (0mM Ca^2+^) | |
| --- | --- | --- |
| Crystal | Form I (0mM Ca^2+^) - 5 | Form I (0mM Ca^2+^) - Merged |
| **Data collection** |  |  |
| Space group | F222 | F222 |
| Wavelength (Å) | 1.7712 | 1.7712 |
| Cell dimensions   |  |  |
| *a*, *b*, *c* (Å) | 126.5, 150.8, 214.2 | 126.5, 150.6, 214.6 |
|     α, β, γ (°) | 90.0, 90.0, 90.0 | 90.0, 90.0, 90.0 |
| Resolution (Å) | 88.3 - 3.8 (4.4 - 3.8) | 39.9 - 3.6 (3.9 - 3.6) |
| *R*_sym_ or *R*_merge_ | 0.142 (0.709) | 0.201 (1.509) |
| *I* / σ*I* | 14.6 (4.1) | 25.2 (5.0) |
| Completeness (%) | 100.0 (100.0) | 100.0 (100.0) |
| Redundancy | 6.8 (6.8) | 34.2 (34.4) |
| No. reflections | 10534 | 12079 |
| Anomalous CC (%) | 14.6 | 25.1 |
| CC_1/2_ (%) | 99.9 (95.8) | 100.0 (97.2) |

Values in parentheses are for highest-resolution shell. CC_1/2_ is defined in reference .

Anomalous CC is the correlation of anomalous differences between random half-sets.

**Table 1 – table source 1. Anomalous data collection**

| Functional State | Inactive (2mM Ca^2+^) | |
| --- | --- | --- |
| Crystal | Form I (2mM Ca^2+^) - 1 | Form I (2mM Ca^2+^) - 2 |
| **Data collection** |  |  |
| Space group | F222 | F222 |
| Wavelength (Å) | 1.7712 | 1.7712 |
| Cell dimensions   |  |  |
| *a*, *b*, *c* (Å) | 126.3, 150.2, 215.1 | 126.0, 150.2, 215.2 |
|     α, β, γ (°) | 90.0, 90.0, 90.0 | 90.0, 90.0, 90.0 |
| Resolution (Å) | 88.2 - 3.2 (3.4 - 3.2) | 88.1 - 3.4 (3.6 - 3.4) |
| *R*_sym_ or *R*_merge_ | 0.070 (1.065) | 0.101 (1.046) |
| *I* / σ*I* | 23.8 (2.4) | 18.1 (2.2) |
| Completeness (%) | 100.0 (100.0) | 98.6 (98.1) |
| Redundancy | 6.8 (6.5) | 6.9 (7.2) |
| No. reflections | 16589 | 13897 |
| Anomalous CC (%) | 32.9 | 15.3 |
| CC_1/2_ (%) | 99.9 (91.8) | 99.9 (93.6) |

| Functional State | Inactive (2mM Ca^2+^) | |
| --- | --- | --- |
| Crystal | Form I (2mM Ca^2+^) - 3 | Form I (2mM Ca^2+^) - 4 |
| **Data collection** |  |  |
| Space group | F222 | F222 |
| Wavelength (Å) | 1.7712 | 1.7712 |
| Cell dimensions   |  |  |
| *a*, *b*, *c* (Å) | 126.2, 150.2, 215.2 | 125.6, 150.0, 215.2 |
|     α, β, γ (°) | 90.0, 90.0, 90.0 | 90.0, 90.0, 90.0 |
| Resolution (Å) | 107.6 - 3.3 (3.5 - 3.3) | 87.9 - 3.2 (3.4 - 3.2) |
| *R*_sym_ or *R*_merge_ | 0.076 (0.991) | 0.072 (1.182) |
| *I* / σ*I* | 21.5 (2.3) | 23.6 (2.4) |
| Completeness (%) | 100.0 (100.0) | 100.0 (100.0) |
| Redundancy | 6.9 (6.8) | 6.8 (6.5) |
| No. reflections | 15080 | 16742 |
| Anomalous CC (%) | 24.1 | 34.4 |
| CC_1/2_ (%) | 99.9 (93.8) | 100.0 (92.8) |

| Ligand-binding State | Inactive (2mM Ca^2+^) | |
| --- | --- | --- |
| Crystal | Form I (2mM Ca^2+^) - 5 | Form I (2mM Ca^2+^) - 6 |
| **Data collection** |  |  |
| Space group | F222 | F222 |
| Wavelength (Å) | 1.7712 | 1.7712 |
| Cell dimensions   |  |  |
| *a*, *b*, *c* (Å) | 125.8, 150.1, 214.8 | 126.2, 150.4, 215.0 |
|     α, β, γ  (°) | 90.0, 90.0, 90.0 | 90.0, 90.0, 90.0 |
| Resolution (Å) | 88.0 - 3.3 (3.4 - 3.3) | 75.2 - 3.3 (3.4-3.3) |
| *R*_sym_ or *R*_merge_ | 0.066 (0.798) | 0.069 (1.142) |
| *I* / σ*I* | 24.4 (2.9) | 23.0 (2.5) |
| Completeness (%) | 99.4 (99.1) | 99.9 (99.9) |
| Redundancy | 6.9 (6.7) | 6.8 (6.6) |
| No. reflections | 15970 | 16132 |
| Anomalous CC (%) | 37.9 | 29.0 |
| CC_1/2_ (%) | 99.9 (95.4) | 99.9 (91.5) |

Values in parentheses are for highest-resolution shell. CC_1/2_ is defined in reference .

Anomalous CC is the correlation of anomalous differences between random half-sets.

**Table 1 – table source 1. Anomalous data collection**

| Functional State | Inactive (2mM Ca^2+^) | |
| --- | --- | --- |
| Crystal | Form I (2mM Ca^2+^) - 7 | Form I (2mM Ca^2+^) - 8 |
| **Data collection** |  |  |
| Space group | F222 | F222 |
| Wavelength (Å) | 1.7712 | 1.7712 |
| Cell dimensions   |  |  |
| *a*, *b*, *c* (Å) | 126.0, 150.3, 215.1 | 126.0, 150.3, 214.9 |
|     α, β, γ  (°) | 90.0, 90.0, 90.0 | 90.0, 90.0, 90.0 |
| Resolution (Å) | 107.6 - 3.2 (3.4 - 3.2) | 88.1 - 3.4 (3.5 - 3.4) |
| *R*_sym_ or *R*_merge_ | 0.068 (1.111) | 0.110 (1.099) |
| *I* / σ*I* | 25.6 (2.5) | 20.2 (2.5) |
| Completeness (%) | 100.0 (100.0) | 100.0 (100.0) |
| Redundancy | 6.8 (6.5) | 6.8 (6.7) |
| No. reflections | 16948 | 14832 |
| Anomalous CC (%) | 34.4 | 21.6 |
| CC_1/2_ (%) | 100.0 (92.7) | 99.9 (93.2) |

| Functional State | Inactive (2mM Ca^2+^) |
| --- | --- |
| Crystal | Form I (2mM Ca^2+^) - Merged |
| **Data collection** |  |
| Space group | F222 |
| Wavelength (Å) | 1.7712 |
| Cell dimensions   |  |
| *a*, *b*, *c* (Å) | 126.0, 150.2, 215.1 |
|     α, β, γ  (°) | 90.0, 90.0, 90.0 |
| Resolution (Å) | 39.8 - 3.2 (3.4 - 3.2) |
| *R*_sym_ or *R*_merge_ | 0.122 (1.502) |
| *I* / σ*I* | 46.4 (6.2) |
| Completeness (%) | 100.0 (100.0) |
| Redundancy | 54.4 (52.6) |
| No. reflections | 17003 |
| Anomalous CC (%) | 58.2 |
| CC_1/2_ (%) | 100.0 (98.8) |

Values in parentheses are for highest-resolution shell. CC_1/2_ is defined in reference .

Anomalous CC is the correlation of anomalous differences between random half-sets.

**Table 1 – table source 1. Anomalous data collection**

| Functional State | Active (10mM Ca^2+^, 10mM L-Trp) | |
| --- | --- | --- |
| Crystal | Form II - 1 | Form II - 2 |
| **Data collection** |  |  |
| Space group | C2 | C2 |
| Wavelength (Å) | 1.7712 | 1.7712 |
| Cell dimensions   |  |  |
| *a*, *b*, *c* (Å) | 107.7, 127.7, 147.0 | 108.1, 127.7, 147.1 |
|     α, β, γ (°) | 90.0, 108.8, 90.0 | 90.0, 108.9, 90.0 |
| Resolution (Å) | 79.7 - 2.7 (3.1 - 2.7) | 139.2 - 2.8 (3.2 - 2.8) |
| *R*_sym_ or *R*_merge_ | 0.043 (0.395) | 0.054 (0.455) |
| *I* / σ*I* | 25.9 / 3.6 | 24.1 / 3.5 |
| Completeness (%) | 97.8 (98.3) | 96.6 (97.6) |
| Redundancy | 3.4 (3.4) | 3.3 (3.4) |
| No. reflections | 48758 | 44316 |
| Anomalous CC (%) | 18.5 | 33.3 |
| CC_1/2_ (%) | 99.9 (98.2) | 99.9 (97.9) |

| Ligand-binding State | Active (10mM Ca^2+^, 10mM L-Trp) | |
| --- | --- | --- |
| Crystal | Form II - 3 | Form II - 4 |
| **Data collection** |  |  |
| Space group | C2 | C2 |
| Wavelength (Å) | 1.7712 | 1.7712 |
| Cell dimensions   |  |  |
| *a*, *b*, *c* (Å) | 107.6, 127.4, 146.6 | 107.7, 127.5, 146.8 |
|     α, β, γ (°) | 90.0, 108.7, 90.0 | 90.0, 108.7, 90.0 |
| Resolution (Å) | 138.8 - 2.7 (3.1 - 2.7) | 139.0 - 2.6 (2.9 - 2.6) |
| *R*_sym_ or *R*_merge_ | 0.051 (0.373) | 0.048 (0.628) |
| *I* / σ*I* | 21.1 / 3.4 | 22.1 / 2.3 |
| Completeness (%) | 97.6 (98.0) | 96.9 (96.0) |
| Redundancy | 3.4 (3.5) | 3.5 (3.5) |
| No. reflections | 48669 | 56415 |
| Anomalous CC (%) | 18.4 | 23.2 |
| CC_1/2_ (%) | 99.9 (98.6) | 99.9 (96.9) |

| Ligand-binding State | Active (10mM Ca^2+^, 10mM L-Trp) |
| --- | --- |
| Crystal | Form II - Merged |
| **Data collection** |  |
| Space group | C2 |
| Wavelength (Å) | 1.7712 |
| Cell dimensions   |  |
| *a*, *b*, *c* (Å) | 107.8, 127.6, 146.9 |
|     α, β, γ (°) | 90.0, 108.8, 90.0 |
| Resolution (Å) | 39.8 - 2.6 (2.7 - 2.6) |
| *R*_sym_ or *R*_merge_ | 0.076 (1.415) |
| *I* / σ*I* | 31.7 / 2.4 |
| Completeness (%) | 99.9 (99.6) |
| Redundancy | 13.4 (13.3) |
| No. reflections | 57732 |
| Anomalous CC (%) | 29.9 |
| CC_1/2_ (%) | 99.9 (99.6) |

Values in parentheses are for highest-resolution shell. CC_1/2_ is defined in reference .

Anomalous CC is the correlation of anomalous differences between random half-sets.

**Table 1 – table source references**

Karplus P A, and Diederichs K. 2012. Linking crystallographic model and data quality*. Scienc***e 3**36: 1030-1033. doi:10.1126/science.1218231.
