# Supplementary material for: Structural mechanism of ligand activation in human calcium-sensing receptor
Source: eLife. 2016 Jul 19;5:e13662. doi: 10.7554/eLife.13662 (PMC4977154; doi:10.7554/eLife.13662)
Supplement: Table 1—source data 2. — DOI: http://dx.doi.org/10.7554/eLife.13662.005 [file elife-13662-table1-data2.doc]

**Table 1 – source data 2**

**Data collection and refinement statistics for endogenous-**

**ligand-bound CaSR ECD**

| Functional State | Active  (10mM Ca^2+^) |
| --- | --- |
| Crystal | Form II |
| **Data collection** |  |
| Space group | C2 |
| Wavelength (Å) | 1.4586 |
| Cell dimensions   |  |
| *a*, *b*, *c* (Å) | 107.4, 127.6, 147.0 |
|     α, β, γ (°) | 90.0, 108.1, 90.0 |
| Resolution (Å) | 79.7 – 2.7 (2.9 – 2.7) |
| *R*_sym_ or *R*_merge_ | 0.051 (0.673) |
| *I* / σ*I* | 12.0 (1.3) |
| Completeness (%) | 92.9 (73.8) |
| Redundancy | 3.4 (3.3) |
| CC_1/2_ (%) | 99.8 (85.4) |
|  |  |
| **Refinement** |  |
| Resolution (Å) | 38.8 – 2.7 |
| No. reflections | 42728 |
| *R*_work_ / *R*_free_ (%) | 20.9 / 23.4 |
| No. atoms |  |
| Protein | 8454 |
| Ligand | - |
| Water | 274 |
| *B*-factors (Å^2^) |  |
| Protein | 63.0 |
| Ligand | - |
| Water | 52.8 |
| R.m.s. deviations |  |
| Bond lengths (Å) | 0.008 |
| Bond angles (°) | 1.14 |

Values in parentheses are for highest-resolution shell.

CC_1/2_ is defined in reference .

**Table 1 – table source references**

Karplus, P.A., and Diederichs, K. 2012. Linking crystallographic model and data quality*. Scienc***e 3**36: 1030-1033. doi:10.1126/science.1218231.
